# Parallel image computation in clusters with task-distributor

**DOI:** 10.1186/s40064-016-2254-x

**Published:** 2016-05-17

**Authors:** Christian Baun

**Affiliations:** Frankfurt University of Applied Sciences, Nibelungenplatz 1, 60318 Frankfurt am Main, Germany

**Keywords:** Cluster computing, Performance, Master–worker scheme, Speedup, POV-Ray

## Abstract

Distributed systems, especially clusters, can be used to execute ray tracing tasks in parallel for speeding up the image computation. Because ray tracing is a computational expensive and memory consuming task, ray tracing can also be used to benchmark clusters. This paper introduces task-distributor, a free software solution for the parallel execution of ray tracing tasks in distributed systems. The ray tracing solution used for this work is the Persistence Of Vision Raytracer (POV-Ray). Task-distributor does not require any modification of the POV-Ray source code or the installation of an additional message passing library like the Message Passing Interface or Parallel Virtual Machine to allow parallel image computation, in contrast to various other projects. By analyzing the runtime of the sequential and parallel program parts of task-distributor, it becomes clear how the problem size and available hardware resources influence the scaling of the parallel application.

## Background

This paper presents the software task-distributor,^1^ which implements the master–worker scheme to simplify the parallel execution of computation of images by using the ray tracing software POV-Ray (Plachetka 1998) in parallel on multiple nodes of a distributed system like a cluster.

Ray tracing is a processor and main memory intensive task, which makes it also useful as benchmark application for clusters. Therefore, task-distributor and POV-Ray can be used to see and understand the impact of the problem size and available main memory resources on the scaling of parallel applications.

This paper is organized as follows. Section “Related work” contains a discussion of related work and explains the reason for the development of task-distributor.

In section “Design decisions”, the general functioning of task-distributor is explained and possible ways to design the software are discussed. The workflow of the software is explained step by step in “Workflow of task-distributor” section.

Section “Parallel image computation inside a cluster” presents a cluster of single board computers. The performance and scalability of this cluster system is analyzed with task-distributor and POV-Ray in “Analysis of performance and scalability” section. Further analysis of the runtime behaviour of task-distributor shows the impact of the problem size and available main memory resources.

Finally, section “Conclusions and future work” presents conclusions and directions for future work.

## Related work

In the past, numerous projects extended POV-Ray in a way that splitting the image computation task into smaller subtasks and distributing them to the nodes of a cluster became possible.

Freisleben et al. (1997) presented a case study of several parallel versions of POV-Ray in a cluster of DEC Alpha workstations by customizing POV-Ray version 3.0 to make use of the MPICH implementation of MPI.^2^ Freisleben et al. (1998) analyzed the runtime behaviour when using up to 11 of the 12 available workstations as worker nodes. It seems that the described solution is no longer available, or has never been released.

Fava et al. (1999) presented MPIPOV,^3^ which extends POV-Ray up to version 3.1 to use the message passing standard MPI. The authors described the speedup by using a cluster of four commodity hardware nodes with two processors per node and compared it to a single SGI Onyx2 workstation, equipped with eight RISC processors.

PVMPOV^4^ is a patch, which extends POV-Ray up to version 3.5 to use PVM.^5^ The latest release of the PVMPOV patch is from the year 2002.

Plachetka (2002) presented a parallel version of POV-Ray version 3.1, which uses PVM. In his work he described the speedup by using up to 48 worker nodes from a cluster with two processors per node.

Yang and Chang (2003) described a Linux cluster scenario, where PVMPOV is used to investigate the speedup.

The described extensions of POV-Ray have not been updated since a decade or longer. Furthermore, they do not support recent versions of POV-Ray. Especially the lack of support for the latest POV-Ray version is a major drawback as POV-Ray supports multithreading since version 3.7. For this reason, the task-distributor software was developed and implemented.

## Design decisions

Task-distributor splits the image calculation by row, which does not require a modification of the POV-Ray source code and no additional library for message passing like MPI or PVM is used. This way, task-distributor largely implements the approach, described by W. R. Rooney^6^ in 2001. By using the POV-Ray options +sr and +er, each node renders just a part (a subset of rows) of the final image. As described in the POV-Ray 3.6 Documentation,^7^ if this is done with POV-Ray 3.6 and older, POV-Ray writes the full height into the image file header, but only the rendered lines into the image.

The parallel approach, described by W. R. Rooney, uses Portable Pixmap (PPM) as output file format and concatenates the resulting parts to the final image. A PPM file header is build and the image parts are assembled via the command line tool cat to compose the final image.

As described in the POV-Ray 3.7 Documentation,^8^ with POV-Ray version 3.7, the output is always a full height image, where the unprocessed rows are filled with black pixels.

Two options for combining the image parts with each other, in order to create the final image, were evaluated during the development of task-distributor:

1. The master node composes the image parts with the command line tool composite from the ImageMagick (Still 2005) project. The following command compares the pixels of the input images and the lighter values are taken for the output image: 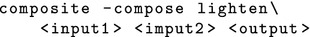 The implementation of this approach is quite simple, but a drawback is, that it is computationally expensive on the master node.
2. The workers remove the unrendered parts by using the command-line tool convert. With this command, a region of ${<}{\texttt {width}}{>}$ by ${<}{\texttt {height}}{>}$ pixels of the image ${<}{\texttt {input}}{>}$, considering the specified horizontal and vertical offsets ${<}{\texttt {offset}}\_{\texttt {x}}{>}$ and ${<}{\texttt {offset}}\_{\texttt {y}}{>}$, is stored in image ${<}{\texttt {output}}{>}$: 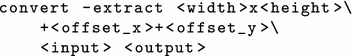 The implementation of this approach is more complex, but advantages of this approach are, that removing the black rows is carried out in parallel by the workers, and the master needs to process lesser data, when it composes (also with convert) the final image from the image parts. As result, the execution time gets reduced.

Because of the described advantages and disadvantages, task-distributor implements the second approach.

Instead of the PPM file format, used by W. R. Rooney, the task-distributor solution uses the raster graphics file format Portable Network Graphics (PNG), which reduces the image size and hence also the load on the master node and the local Ethernet. While PPM is a very simple file format, that does not implement any sort of compression functionality, PNG implements the lossless compression method deflate.^9^ Therefore, storing an image in the file format PNG, instead of PPM, significantly reduces the file size without losing quality. The exact file size and compression ratio depends of the number of pixels, color depth and image content. As described by Roelofs (1999), the only convincing way to demonstrate the compression benefits of one image format over another is to do an comparison of the two on a set of real images. Table 1 shows a comparison of the file size of the example scene blob.pov in file format PPM and file format PNG as well as the compression ratio. This scene was also used for analyzing the performance and scalability of a cluster system with task-distributor and POV-Ray (see “Analysis of performance and scalability” section).

**Table 1:** File size of the example scene blob.pov, rendered in different resolutions and stored in the file formats PPM and PNG, as well as the compression ratio

Resolution	PPM file size (Bytes)	PNG file size (Bytes)	Compression ratio
200 × 150	90,142	8974	≈10
400 × 300	360,142	24,994	≈14
800 × 600	1,440,142	67,159	≈21
1600 × 1200	5,760,144	184,827	≈31
3200 × 2400	23,040,144	519,951	≈44
6400 × 4800	92,160,144	1,451,245	≈63

The compression ratio is the ratio between the uncompressed size (PPM) and compressed size (PNG)

## Workflow of task-distributor

A shared folder, accessible by the master and the workers, must be created. It is used to store the lockfile and the image parts and can be implemented by using a distributed file system or a protocol like the Network File System (NFS).

**Fig. 1:**
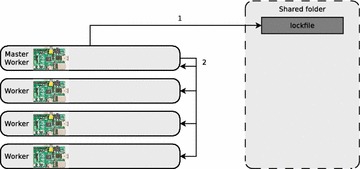
The master creates a lockfile and starts ray tracing jobs

First the master creates a lockfile on the shared folder. Then the master starts a POV-Ray task on each worker node via secure shell (see Fig. 1). The task-distributor implements Round Robin load balancing, which does not take the load of the nodes into account. This is not a problem, as long as the cluster is a homogeneous^10^ one and the cluster nodes are all used for the same tasks.

**Fig. 2:**
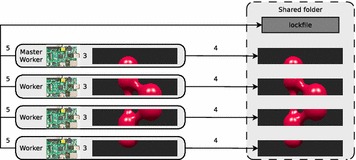
The workers calculate their image parts, copy them into the shared folder and insert their hostnames into the lockfile

At step three (see Fig. 2), the workers calculate the assigned image parts and remove the black rows via the convert tool. This step is executed in parallel on all worker nodes. After the POV-Ray jobs have been started, the master checks in an infinite loop the lockfile to determine the execution status of the workers.

After a worker has finished calculating its assigned job, it copies the result into the shared folder (step four) and writes its hostname into the lockfile (step five). Both steps are executed in parallel on all worker nodes. The distributed file system or protocol used prevents data corruption, caused by parallel write operations of the workers.

**Fig. 3:**
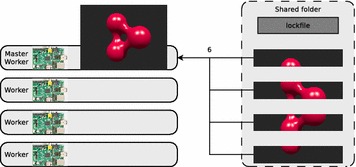
The master creates the final image

**Fig. 4:**
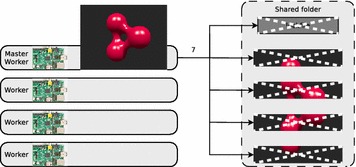
The master cleans up the shared folder

In step six, the master sequentially composes the image parts by using the convert tool to create the final image (see Fig. 3). At the final step seven, the master erases the lockfile and the image parts from the shared folder (see Fig. 4). As for step six, this task cannot be parallelized.

## Parallel image computation inside a cluster

**Fig. 5:**
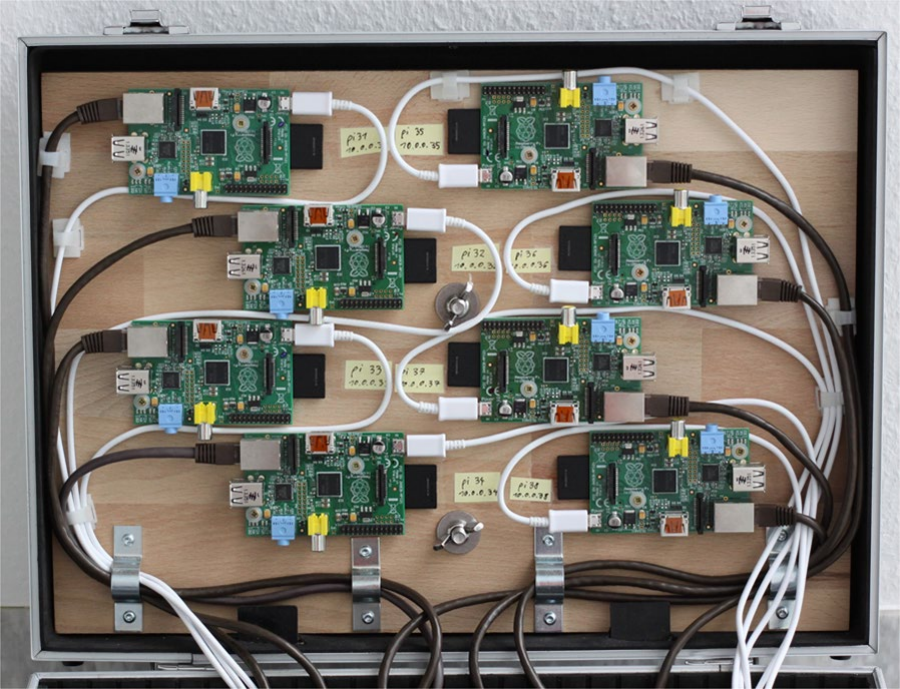
Eight Raspberry Pi Model B are the cluster nodes

**Fig. 6:**
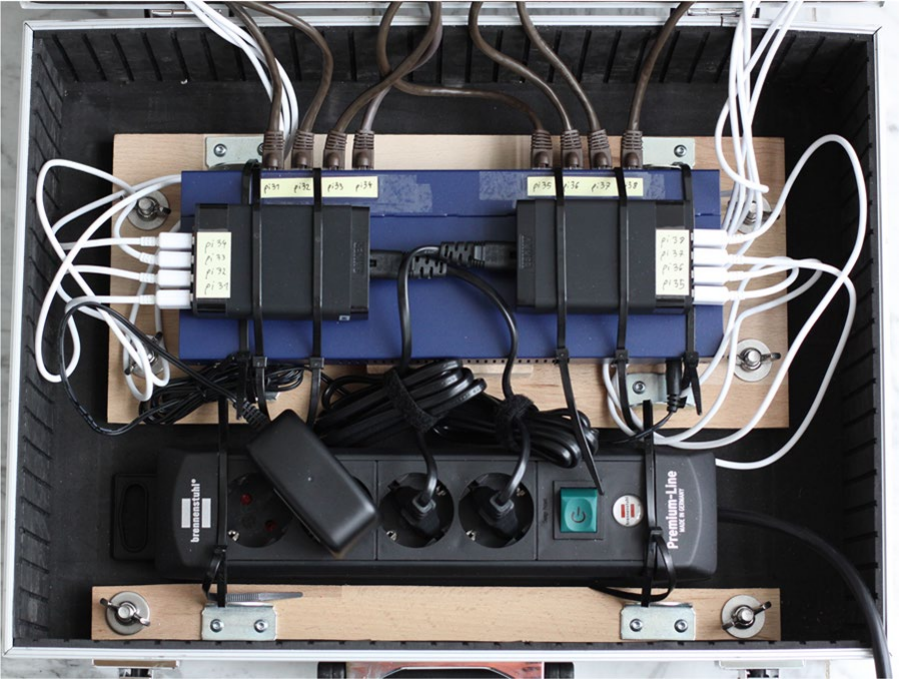
Power supply and network infrastructure of the cluster

In order to show how task-distributor and POV-Ray can be used to analyze the performance of a distributed system, a cluster (see Figs. 5, 6) of the following components was constructed:

- 8× Raspberry Pi Model B single board computer
- 8× SD flash memory card (16 GB each)
- 10/100 network switch with 16 ports
- 8× network cable CAT 5e U/UTP
- 2× USB power supply 40 W (5 V, 8 A)
- 8× USB 2.0 cable USB-A/Micro-USB

The nodes are single-processor systems with an ARM 11 CPU equipped with 512 MB main memory. Increasing the clock rate of a Raspberry Pi from 700 to 800 MHz does not require to overvolt the CPU and results in a noticeable increase of processing power and was therefore used in all tests.

The purchase cost for all components were approximately 500 €. The throughput of a 100 Mbit Ethernet switch is sufficient for Raspberry Pi computers in line with their standard 100 Mbit Ethernet interface.

A cluster of single board computers has very limited resources and cannot compete with the performance of higher-value systems. But despite these drawbacks, it is a promising and economic option for academic purposes like student projects or research projects with limited financial resources.

Another advantage of such a cluster system is the power consumption of the cluster, which is just ≈24 W in idle operation mode and ≈26 W in stress mode.^11^

## Analysis of performance and scalability

Like every parallel program, task-distributor consists of sequential and parallel parts. To understand its scalability in the evaluated cluster of Raspberry Pi single board computers, task-distributor was used to compute the example scene blob.pov, which is included in POV-Ray version 3.7. The scene was computed with different numbers of nodes in different resolutions. Each increase of the resolution results in four times as many pixels as with the resolution before. It is of particular interest how the limited hardware resources, especially the available main memory, influences the performance of the cluster.

**Fig. 7:**
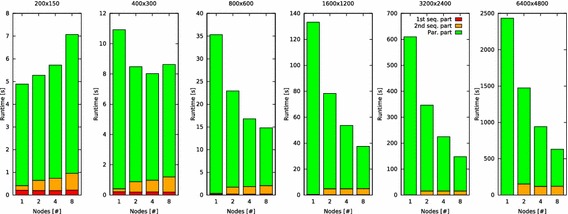
Total runtime of task-distributor while ray tracing an image (s)

 The results presented in Figs. 7, 9 and 10 are average values of ten test cycles.

### Analysis of the runtime

The diagrams in Fig. 7 show the total runtime of task-distributor. The runtimes of the sequential and parallel parts are highlighted with different colors. The steps, which are carried out during the first sequential part are explained in Fig. 1. The steps of the parallel part are shown Fig. 2. Figures 3 and 4 present the steps of the second sequential part.

**Table 2:** Runtime of the sequential and parallel parts of task-distributor in the cluster of Raspberry Pi computers when a single one, two, four or eight nodes (processors) are used

	Resolution	1st seq. part (s)	2nd seq. part (s)	Parallel part (s)
1 node used	200 × 150	0.210	0.204	4.472
	400 × 300	0.215	0.202	10.505
	800 × 600	0.206	0.194	34.918
	1600 × 1200	0.206	0.205	132.840
	3200 × 2400	0.242	0.233	609.427
	6400 × 4800	0.219	0.468	2434.630
2 nodes used	200 × 150	0.200	0.449	4.628
	400 × 300	0.199	0.680	7.603
	800 × 600	0.207	1.552	21.167
	1600 × 1200	0.217	4.665	73.481
	3200 × 2400	0.203	15.243	331.059
	6400 × 4800	0.211	*152.037*	1321.640
4 nodes used	200 × 150	0.198	0.545	4.984
	400 × 300	0.207	0.777	7.043
	800 × 600	0.203	1.632	14.945
	1600 × 1200	0.243	4.625	48.780
	3200 × 2400	0.230	15.146	209.375
	6400 × 4800	0.219	*120.324*	821.244
8 nodes used	200 × 150	0.216	0.741	6.115
	400 × 300	0.201	1.002	7.422
	800 × 600	0.242	1.839	12.727
	1600 × 1200	0.204	4.802	32.458
	3200 × 2400	0.218	15.543	131.780
	6400 × 4800	0.209	*120.471*	509.707

All values in the table are rounded to three decimal places behind the decimal point

Table 2 contains the measurement values that were used to create the diagrams in Fig. 7.

For almost all tested resolutions (except 200 × 150 and 400 × 300 pixels) applies the rule that additional nodes reduce the total runtime. When the image is computed with resolution 200 × 150, not only the runtime of the second sequential part increases when the number of nodes grows, but also the runtime of the parallel part. This implies that the problem size is too small to compute it in parallel efficiently.

During the execution of the parallel part, the nodes compute the image parts in parallel by using POV-Ray. The size $S_{B}$ of the temporary buffer of POV-Ray is calculated by Eq. (1) for a specific $XY$ resolution.

Table 3 contains the size of the temporary buffer for the tested resolutions of Fig. 7.

1 $$S_{B} = X \times \ Y \times \ {\texttt {sizeof(double)}} \times \ 5\,\text{ Bytes }$$

The size of the data type double is 8 Bytes. The reason for the multiplication by 5 Bytes is because POV-Ray implements a 5-channel color model^12^ with a single byte for each channel.

**Table 3:** Size of the temporary buffer of POV-Ray

Resolution	Buffer size (Bytes)
200 × 150	1,200,000
400 × 300	4,800,000
800 × 600	19,200,000
1600 × 1200	76,800,000
3200 × 2400	307,200,000
6400 × 4800	1,228,800,000
12800 × 9600	4,915,200,000

For resolution 400 × 300, the temporary buffer of POV-Ray is 4,800,000 Bytes in size. Due to the small problem size, using eight nodes instead of four nodes does not reduce, but increase the required runtime.

**Fig. 8:**
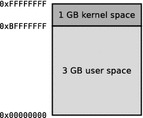
Virtual memory address-space layout of 32-bit Linux operating systems with kernel space and user space

Efforts have been made to investigate the runtime for resolution 12800 × 9600, but all attempts resulted in an immediate program termination of POV-Ray. This is caused by the 32-bit-architecture of the Raspberry Pi computer. The required temporary buffer for resolution 12800 × 9600 is 4,915,200,000 Bytes (see Table 3) in size and this exceeds the size of the user space^13^ of a 32-bit operating system (see Fig. 8).

A notable observation, which can be seen in the measurement values in Table 2 is the significant increase of the runtime of the second sequential part for resolution 6400 × 4800 compared with resolution 3200 × 2400 (see the italicized values in Table 2). While the number of pixels gets just quadrupled, the runtime increases by factor 8–10. The cause of this phenomenon is explained in “Analysis of the percentages of the sequential and parallel parts of the runtime” section.

### Analysis of the speedup

**Fig. 9:**
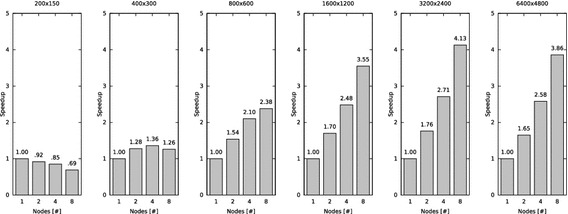
Speedup when task-distributor is used to ray trace an image

The diagrams in Fig. 9 show the speedup of task-distributor. The speedup $S_{P}$, that can be achieved when running a program on $P$ processors is defined as:

2 $$S_{P} = \frac{T_{1}}{T_{P}}$$

where $T_{1}$is the runtime on a single-processor system and $T_{P}$ is the runtime on a multiprocessor system.

The theoretical maximum speedup^14^$S_{T}$ is equal to the number of single-processor nodes.

The results show again that with resolution 200 × 150 the problem size is too small to be efficiently computed in parallel—increasing the number of nodes (processors) decreases the speedup. Calculating the image with a resolution of 400 × 300 increases the problem size. Thus, the program can be parallelized more efficiently, yet the speedup is significantly worse compared to the theoretical maximum speedup.

The diagrams for a resolution of 800 × 600, 1600 × 1200 and 3200 × 2400 show that the more the problem size increases, the better are the results are when the program is executed in parallel. Consequently, with each enlargement of the problem size (resolution), the speedup gets closer to the theoretical maximum speedup.

The measurement results of resolution 6400 × 4800 are worse compared to the results of resolution 3200 × 2400. Even though the resolution 6400 × 4800 increases the problem size further, the speedup trend with an increasing number of nodes is not as good as compared with the resolution 3200 × 2400. To analyze this phenomenon, the percentages of the sequential and parallel parts of the runtime are examined.

### Analysis of the percentages of the sequential and parallel parts of the runtime

**Fig. 10:**
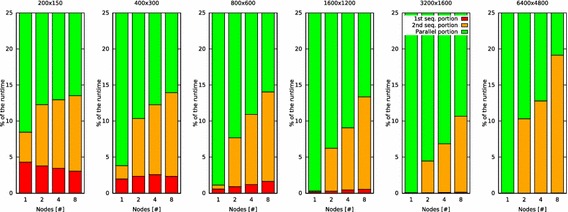
Percentages of task-distributors’ sequential and parallel parts of the runtime while ray tracing an image (%)

The diagrams in Fig. 10 show the percentages of the sequential and parallel parts of the runtime of task-distributor. Table 4 contains the measurement values that were used to create the diagrams in Fig. 10.

The values show that regardless of the number of nodes used, increasing the problem size by increasing the resolution results in a reduction of the percentage of the sequential parts runtime, except for resolution 6400 × 4800. With this resolution, the percentage of the second sequential part significantly raises and consequently the percentage of the parallel part declines.

**Table 4:** Proportions of sequential and parallel parts of task-distributors’ runtime in the cluster of Raspberry Pi computers when a single one, two, four or eight nodes (processors) are used

	Resolution	1st seq. part (%)	2nd seq. part (%)	Parallel part (%)
1 node used	200 × 150	4.29	4.16	91.55
	400 × 300	1.96	1.84	96.20
	800 × 600	0.58	0.54	98.88
	1600 × 1200	0.15	0.15	99.70
	3200 × 2400	0.03	0.03	99.94
	6400 × 4800	^a^	0.01	99.99
2 nodes used	200 × 150	3.78	8.49	87.73
	400 × 300	2.34	8.00	89.66
	800 × 600	0.90	6.76	92.34
	1600 × 1200	0.27	5.95	93.78
	3200 × 2400	0.05	4.39	95.56
	6400 × 4800	0.01	*10.31*	*89.68*
4 nodes used	200 × 150	3.44	9.51	87.05
	400 × 300	2.57	9.67	87.76
	800 × 600	1.20	9.72	89.08
	1600 × 1200	0.45	8.62	90.93
	3200 × 2400	0.10	6.73	93.17
	6400 × 4800	0.02	*12.77*	*87.21*
8 nodes used	200 × 150	3.04	10.47	86.49
	400 × 300	2.32	11.61	86.07
	800 × 600	1.63	12.41	85.96
	1600 × 1200	0.54	12.81	86.65
	3200 × 2400	0.14	10.53	89.33
	6400 × 4800	0.03	*19.11*	*80.86*

^a^ The value is too small for representing it in this table

This phenomenon is caused by the amount of free main memory on the single nodes in the cluster. The most resource consuming task of the second sequential part of task-distributor execution is the composing of the image parts via convert to create the final image. This task (see Fig. 3) is carried out by the master and it cannot be executed in parallel.

Each one of the Raspberry Pi cluster nodes is equipped with 512 MB main memory. A part of the main memory is assigned as video memory to the GPU, which lacks own dedicated memory. Because in the cluster, the GPUs are not used at all, the minimal GPU memory was set, which is 16 MB. This results in 496 MB main memory left for the operating system and the applications on each node. After the operating system Raspbian and the daemon and client for the distributed file system is started, approx. 400–450 MB main memory remains available on each node.

The image parts created by POV-Ray (see Fig. 2) are stored in the file format PNG and the convert tool (see Fig. 3) uses the same file format to store the final image. The amount of main memory $M$, which needs to be allocated by convert for creating the final image depends on the number of channels per pixel $C$, the number of bits per pixel channel $B$ and the resolution $XY$ of the input and output images and is calculated with Eq. 3.

3 $$M = X \times \ Y \times \ B \times \ C$$

ImageMagic version 6.7.7 was used for this project. This software allocates 16 bits per pixel channel and four channels per pixel. Therefore, the required memory per pixel is $16 * 4 = 64$ bits. As convert needs to allocate memory for the output as well as for the input images, it allocates at least double the amount of $M$. Table 5 shows the calculated minimal memory consumption for different resolutions. In practice, convert allocates approx. 3–5 MB (depending on the number of input files) additional main memory for the application itself.

**Table 5:** Minimal memory consumption of convert

Resolution	Minimal memory consumption (Bytes)
200 × 150	480,000
400 × 300	1,920,000
800 × 600	7,680,000
1600 × 1200	30,720,000
3200 × 2400	122,880,000
6400 × 4800	491,520,000

When convert concats the image parts to create the final image, the memory of one Rasperry Pi node is sufficient. But in case of a resolution of 6400 × 4800, the required minimum main memory exceeds the available free main memory and a temporary file is created by convert on the file system.

Because the temporary file resides outside the main memory, e.g. on the (micro-)SD storage, the runtime of convert increases due to a lower IO performance compared to the main memory (see the italicized values for the second sequential part in Table 4).

## Conclusions and future work

The parallel image computation by using POV-Ray in clusters can be simplified with the task-distributor software solution. In contrast to the existing solutions, described in “Related work” section, the task-distributor solution does not require a message passing system like MPI or PVM and no modification of the POV-Ray source code is necessary. In addition, it utilizes more efficiently the existing network resources and as much computational effort as possible is carried out in parallel by the workers.

Clusters of single board computers like the Raspberry Pi are useful for academic purposes and research projects because of the lesser purchase- and operation costs compared to commodity hardware server resources.

Analyzing the runtime and speedup of task-distributor could show that a cluster of single board computers is an appropriate platform to see and understand the scaling of parallel applications, the influence of the problem size and the impact of the available main memory resources.

Task-distributor can be adapted with little effort in a way that it executes not only POV-Ray jobs, but also any other application in parallel in distributed systems.

A useful enhancement of task-distributor, especially for heterogeneous clusters, would be the implementation of a load balancing functionality, that takes the state and load of the single nodes into account. The load could be measured with solutions like Ganglia (Massie et al. 2004) or Nagios (Barth 2008). The acquired load information could be used as basis for the scheduling of the single subtasks.

Further next steps are the implementation of clusters of different single board computers like the BananaPi or ODROID-U3 and comparing their performance.

Since February 2015, the Raspberry Pi 2 is available and provides more computational power and main memory compared to the cluster nodes in this study. Building a cluster of this computers is one of the next steps. It is interesting to discover how increasing the processor cores by factor four and doubling the main memory per node affects the runtime and speedup of task-distributor because the available main memory per processor core is halved.

## Footnotes

1 Further information about the task-distributor software, including the source code, can be found at the web page http://github.com/christianbaun/task-distributor/.

2 The Message Passing Interface (MPI) it is the de facto standard for distributed scientific computing and defines the syntax and semantics of the MPI functions. MPICH is one of several implementations of the standard.

3 Further information about the MPI patch for POV-Ray provides the web page http://www.ce.unipr.it/research/parma2/povray/povray.html.

4 Further information about the PVM patch for POV-Ray provides the web page http://pvmpov.sourceforge.net.

5 The Parallel Virtual Machine (PVM) is a software, which allows to connect heterogeneous computer systems to a single distributed parallel computer. Since the late 1990s, PVM is more and more superseded by MPI.

6 Further information about the approach for using POV-Ray inside a Cluster, described by W. R. Rooney in 2001, provides the web page http://homepages.ihug.co.nz/~wrooney/present/povclust.html.

7 See the corresponding section of the POV-Ray 3.6 documentation on web page http://www.povray.org/documentation/view/3.6.0/217/.

8 See the corresponding section of the POV-Ray 3.7 documentation on web page http://www.povray.org/documentation/3.7.0/r3_2.html.

9 Further information about the deflate algorithm provides the web page http://www.zlib.net/feldspar.html.

10 In a homogeneous cluster, all nodes consist of the same hardware components and run the same operating system.

11 The nodes were put into stress mode by using the command-line tool stress. Further information about stress provides the web page http://people.seas.harvard.edu/~apw/stress/.

12 The channels are *red*, *green*, *blue*, *filter* and *transmit*. While *filter* specifies the amount of filtered transparency of a substance, *transmit* specifies the amount of non-filtered light, which is transmitted through a surface.

13 The virtual memory address space of a 32-bit operating system is $4 \times 2^{30}$ Bytes in size. A single process cannot occupy more than the $3 \times 2^{30}$ Bytes of the so called user space of its virtual memory. The upper part of the address space is called kernel space and is used only by the operating system.

14 The theoretical maximum speedup $S_{T}$ is value 2 for two nodes, value 4 for four nodes, value 8 for eight nodes, etc.
